# Nobiletin Ameliorates the Deficits in Hippocampal BDNF, TrkB, and Synapsin I Induced by Chronic Unpredictable Mild Stress

**DOI:** 10.1155/2013/359682

**Published:** 2013-03-17

**Authors:** Jing Li, Ying Zhou, Bin-Bin Liu, Qing Liu, Di Geng, Lian-Jin Weng, Li-Tao Yi

**Affiliations:** ^1^Department of Chemical and Pharmaceutical Engineering, College of Chemical Engineering, Huaqiao University, Xiamen, Fujian 361021, China; ^2^Department of Biotechnology and Bioengineering, College of Chemical Engineering, Huaqiao University, Xiamen, Fujian 361021, China

## Abstract

*Background*. Our previous study has demonstrated that nobiletin could reverse the behavioral alterations in stressed mice. However, the relation of its antidepressant-like action with neurotrophic molecular expression remains unknown. This study aimed to explore the antidepressant-like mechanism of nobiletin related to the neurotrophic system in rats exposed to chronic unpredictable mild stress (CUMS). *Methods*. Depressive-like anhedonia (assessed by sucrose preference) and serum corticosterone secretion were evaluated in the CUMS, followed by brain-derived neurotrophic factor (BDNF), its tropomyosin-related kinase receptor B (TrkB), and the downstream target synapsin I expressions in the hippocampus. *Results*. Anhedonia, which occurred within week 2, was rapidly ameliorated by nobiletin. While fluoxetine needed additional 2 weeks to improve the anhedonia. In addition, nobiletin administration for 5 weeks significantly ameliorated CUMS-induced increase in serum corticosterone levels. Furthermore, we also found that CUMS-induced deficits of hippocampal BDNF, TrkB, and synapsin I were ameliorated by nobiletin. *Conclusions*. Taken together, these findings suggest that nobiletin produces rapidly acting antidepressant-like responses in the CUMS and imply that BDNF-TrkB pathway may play an important role in the antidepressant-like effect of nobiletin.

## 1. Introduction

Depression, one of the major causes of disability worldwide, is a mood or affective disorder caused by many factors, from the psychological, social, and environmental to the genetic, and metabolics [1]. Although clinical and experimental researches have provided some insight into the pathophysiological processes that may be occurring in the depression; at present, the molecular correlates, underpinning these abnormalities, are not fully understood [2]. Neurotrophins are a family of proteins that play multiple roles in regulating neural survival, development, function, and plasticity [3]. Limiting quantities of neurotrophins control the numbers of surviving neurons to ensure a match between neurons and the requirement for a suitable density of target innervation [4]. Beyond the promotion of neural function, the family is also a powerful modulator in hippocampal-dependent learning and memory [5]. Brain-derived neurotrophic factor (BDNF), one of the most widely distributed neurotrophins, after binding with and activating tropomyosin-related kinase receptor B (TrkB), is involved in the pathophysiology and treatment of depression [6, 7]. They play a critical role in the modulation of some functions such as neurotransmitter release and postsynaptic responses to neurotransmitters, which are closely related to the antidepressant therapy [8]. Either reduced BDNF availability or decreased expression of TrkB receptor could reduce BDNF-TrkB signaling in animals [9]. In contrast, treatment with antidepressants could upregulate BDNF or activate TrkB receptor in the brain in rodents [10, 11]. Additionally, in the animal model of depression, results demonstrate that antidepressant efficacy is mediated at least in part through an elevation of BDNF levels or BDNF-TrkB signaling in the hippocampus [12, 13]. On the contrary, in transgenic animals with decreased brain BDNF levels or inhibited BDNF-TrkB signaling, antidepressant agents fail to exert behavioral responses [14, 15]. Collectively, the findings reviewed earlier strongly suggest that the behavioral effects of antidepressants require functional BDNF signaling in the brain.

Stress is an important precipitant factor in depression, and the changes in various body systems that occurred in depression are similar to those observed in response to stress [16]. Chronic unpredictable mild stress (CUMS), the most promising rodent model for depression, is widely used for preclinical testing and screening of antidepressants [17]. In the CUMS paradigm, animals are subjected to a variety of mild stressors presented intermittently for prolonged periods of time, which mimic chronic stressful life events and result in a behavioral deficit anhedonia, a core symptom of human depression [18]. Anhedonia is monitored by a reduction in sucrose preference, which can be restored by therapeutically effective drugs for the treatment of depression [11].

Nobiletin, a dietary constituent of flavonoids isolated from citrus fruits, has been reported to upregulate synaptic transmission and improve memory impairment in rodents [19, 20]. Our previous study found that nobiletin administration significantly reduced the immobility time in both the tail suspension test (TST) and forced swimming test (FST) without accompanying changes in locomotor activity in the open-field test (OFT) in mice [21]. Furthermore, we also demonstrated that the antidepressant-like effect of nobiletin was mediated by monoaminergic systems in this study. However, until now, the neurotrophic molecular expression underlying the antidepressant-like effect of nobiletin remains unknown.

Therefore, considering that hippocampus is critical for stress, learning, and memory processes in depression and in the antidepressant response to pharmacotherapy [22–24], the aim of the present study was to evaluate the effects of daily nobiletin treatment on sucrose preference and corresponding changes in BDNF, TrkB receptor, and the downstream target synapsin I in the hippocampus after CUMS.

## 2. Materials and Methods

### 2.1. Animals

Male Sprague-Dawley rats (260–300 g) were purchased from Laboratory Animal Centre, Fujian Medical University, Fujian Province, China. Animals were single-housed under a normal 12-h/12-h light/dark cycle with the lights on at 07:00 AM. Ambient temperature and relative humidity were maintained at 22 ± 2°C and at 55 ± 5%, and animals were given a standard chow and water *ad libitum* for the duration of the study, except when the CUMS procedure or sucrose preference test was required. The animals were allowed to acclimatize to these conditions for 1 week before any experimental procedure was initiated. At the beginning of the experiments the average body weight of rats was approximately 310 g. All procedures were performed in accordance with the published guidelines of the China Council on Animal Care (regulations for the Administration of Affairs concerning experimental animals, approved by the State Council on October 31, 1988, and promulgated by Decree no. 2 of the State Science and Technology Commission on November 14, 1988).

### 2.2. Chemicals and Reagents

Nobiletin (purity >98% by HPLC) was obtained from Shanxi Huike Botanical Development Co., Ltd. (Xi'an, China). Fluoxetine hydrochloride was purchased from Changzhou Siyao Pharmaceuticals Co., Ltd. (Changzhou, China). All primers used in this study were designed and synthesized by Sangon Biotech Co. Ltd. (Shanghai, China). The anti-BDNF (Catalog number: sc-546, detecting mature BDNF) and anti-TrkB (Catalog number: sc-12, detecting full-length TrkB) antibody and the respective secondary antibodies were purchased from Santa Cruz Biotechnology Inc. (Santa Cruz, USA). The antisynapsin I (Catalog number: AB1543, detecting synapsin I) antibody was purchased from Millipore Corporation (Billerica, USA). The anti-GAPDH (Catalog number: KC-5G5, detecting GAPDH) antibody was purchased from Kangcheng Biotech (Shanghai, China). Trizol reagent was purchased from Invitrogen (Carlsbad, USA). Reverse transcriptase moloney murine leukemia Virus (M-MLV) used for cDNA synthesis was from Promega Corporation (Madison, USA). All other reagents used in polymerase chain reaction (PCR) and western blot were purchased from Sangon Biotech Co. Ltd. (Shanghai, China).

### 2.3. CUMS Procedure

The CUMS procedure was performed according to the traditional method described by Willner et al. [17], with some modifications. Briefly, the weekly stress regime consisted of food and water deprivation, stroboscopic illumination (150 flashes/min), white noise, light/dark succession every 2 h, overnight illumination, 45° cage tilt, soiled cage, and pair-housing (Table 1). All of the stressors which were applied individually and continuously were randomly scheduled over a 1-week period and repeated throughout the 5-week procedure. The control group was housed in a separate room and had no contact with the stressed animals. These rats were deprived of food and water for the 18 h preceding each sucrose test, but otherwise food and water were freely available in the home cage. 

### 2.4. Sucrose Preference Test

Before the beginning of CUMS procedure, all rats were given 1% sucrose solution for 24 hours. Then, both sucrose solution and fresh water were made accessible to the rats for another 24 hours. After deprived of drinking for 23 hours, the rats were given both 1% sucrose solution and fresh water for 1 hour again. After this sucrose consumption training phase, the animals were randomly divided into 5 groups (8 rats per group): control-vehicle (saline), CUMS-vehicle (saline), CUMS-fluoxetine (positive control, 10 mg/kg), and CUMS-nobiletin at a dose of 20 mg/kg and 40 mg/kg. Nobiletin was suspended in saline with 10% (v/v) Tween-80 (polyoxyethylene sorbitan monooleate), and fluoxetine hydrochloride was dissolved in 0.9% physiological saline. Four groups except control-vehicle were exposed to the CUMS procedure for 5 weeks and treated. All drugs were administered by gavage in a volume of 10 mL/kg body once daily at 11:00 AM for 5 weeks. Through the period of CUMS and treatment, sucrose preference test were conducted following an 18-h food and water deprivation at 11:00 AM every Tuesday. Sucrose preference was calculated as sucrose preference (%) = sucrose intake (mL)/[sucrose intake (mL) + water intake (mL)] × 100%. The treatment protocol of dose and administration route used for nobiletin and fluoxetine was adopted according to our and other previous studies, and was calculated based on the body surface area of the rat [11, 21].

### 2.5. Blood Sampling and Tissue Extraction

After the last sucrose preference, all rats were decapitated between 12:00 PM (midday) and 2:00 PM to avoid fluctuation of hormone levels. Blood samples were collected immediately. The brain region of hippocampus was isolated immediately, and then stored at −80°C for later analysis of mRNA and protein levels.

### 2.6. Serum Corticosterone Assay

Blood was collected on ice and separated in a refrigerated centrifuge at 4°C (4000 ×g for 10 min). Serum was stored at –20°C until assays were performed. Serum corticosterone levels were measured using a commercial kit (Enzo Life Sciences, Plymouth Meeting, USA) based on enzyme immunoassay.

The concentrations of corticosterone in each sample were determined in duplicate, and the average of the two values was used as the value for that rat.

### 2.7. Real-Time PCR

Total RNA was extracted from hippocampus using Trizol reagent following the manufacturer's instructions. Reverse transcription was performed using M-MLV reverse transcriptase for cDNA synthesis. Real-time PCR reactions were performed using a SYBR Premix Ex Taq Kit in ABI-7500 system. The BDNF (NM_012513; sense primer 5′-TGTGACAGTATTAGCGAGTGGGT-3′ and antisense primer 5′-CGATTGGGTAGTTCGGCATT-3′), TrkB (NM_012731; sense primer 5′-CTTATGCTTGCTGGTCTTGG-3′ and antisense primer 5′-GGGTATTCTTGCTGCTCTCA-3′), synapsin I (NM_019133; sense primer 5′-CCCTTCATTGATGCTAAATACG-3′ and antisense primer 5′-GTTGACCACAAGTTCCACGAT-3′), and the internal control GAPDH (NM_017008; sense primer 5′-ACCACAGTCCATGCCATCAC-3′ and antisense primer 5′-TCCACCACCCTGTTGCTGTA-3′) primers were used. The fluorescence signal was detected at the end of each cycle. Melting curve analysis was used to confirm the specificity of the products. The results were analyzed by the 2^−ΔΔCT^ method.

### 2.8. Western Blot

Brain samples were homogenized in a lysis buffer containing 50 mM Tris-HCl (pH7.4), 1 mM EDTA, 150 mM NaCl, 1% Triton X-100, 1% sodium deoxycholate, 0.1% SDS, 1 mM trichostatin A, and phosphatase inhibitor cocktail. The homogenates were centrifuged at 14000 ×g for 20 min at 4°C, and the supernatants were collected. The protein concentration was determined by a BCA assay. Total proteins were separated by SDS-PAGE and transferred to a PVDF membrane. Following blocking in 3% BSA/TBST at room temperature for 1 h, the membranes were incubated with the appropriate primary antibodies at 4°C overnight (anti-BDNF: 1 : 500, anti-TrkB: 1 : 1000, antisynapsin I: 1 : 1000, and anti-GAPDH: 1 : 5000). After being washed with TBST for three times, the membranes were incubated with an HRP-labeled secondary antibody (1 : 4000). The blots were washed again for three times by TBST buffer, and the immunoreactive bands were detected by using the enhanced chemiluminescence method. Western blot bands were scanned by Hewlett-Packard Scanjet 5590 and subsequently analyzed densitometrically with Bio-Rad Quantity One software. The results were normalized using GAPDH expression as the internal standard.

### 2.9. Statistical Analyses

All data were expressed as mean ± SEM. SPSS software (version 13.0) was used for statistical analyses. Data of sucrose preference and body weight from the CUMS were analyzed using a repeated ANOVA with treatment as between factor and time (weeks) as within factor. For biochemical analysis, a one-way ANOVA followed by post-hoc Dunnett's test was performed. A value of *P* < 0.05 was considered statistically significant for analysis. The figures were obtained by GraphPad Prism (version 5).

## 3. Results

### 3.1. Effects of Nobiletin on the Sucrose Preference and Body Weight Gain in the CUMS

**Figure 1:**
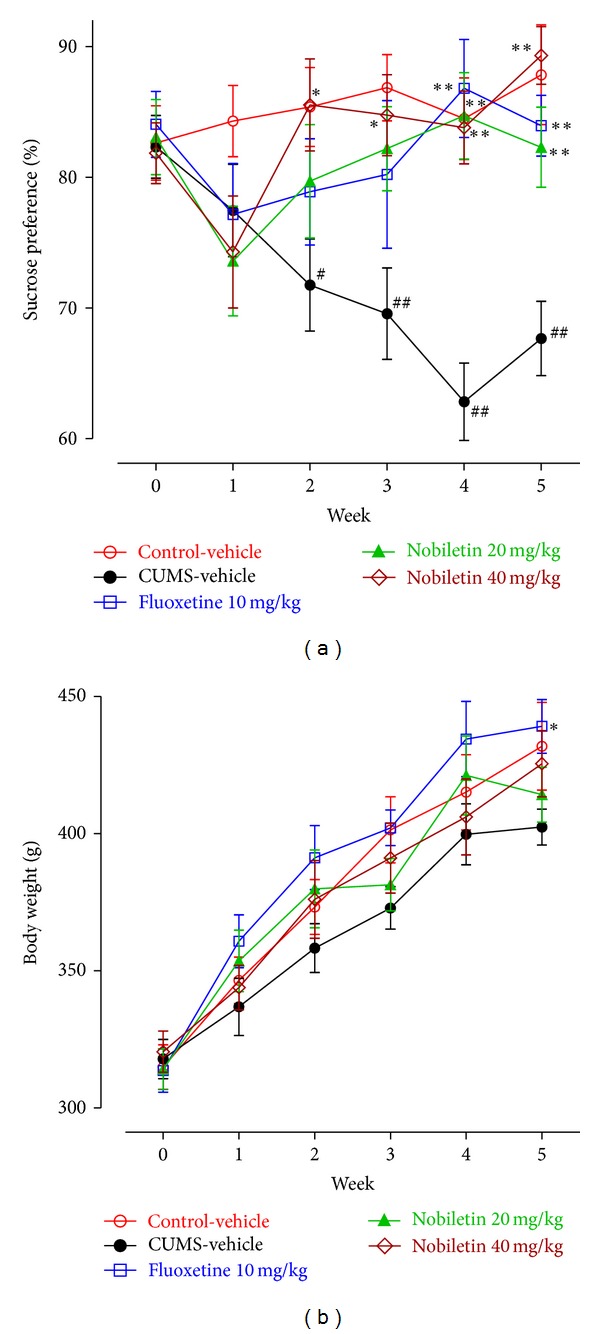
Effects of nobiletin on the sucrose preference (a) and body weight (b) in CUMS-induced rats. The data represented the values of mean ± SEM (*n* = 8). ^#^ *P* < 0.05 and ^##^ *P* < 0.01 versus control-vehicle group. *P < 0.05 and **P < 0.01 versus CUMS-vehicle group.

Figure 1(a) presented the sucrose preference in rats at baseline (week 0) and during a 5-week CUMS period. Separated one-way ANOVA revealed no statistical significance effect on sucrose preference (*F*(4,35) = 0.14, *P* > 0.05) among the groups on the baseline test. A repeated ANOVA with treatment as independent factor and week as repeated factor, revealed that there was a statistically significant effect of treatment (*F*(3,28) = 13.92, *P* < 0.01) and treatment × week interaction (*F*(15,140) = 2.27, *P* < 0.01), but not effect of week (*F*(5,140) = 1.90, *P* > 0.05) on the sucrose preference.

Furthermore, the sucrose preference of CUMS-vehicle rats was significantly lower compared to control-vehicle animals, at weeks 2, 3, 4, and 5 (*F*(1,14) = 8.60; 16.03; 25.31; 17.98; *P* < 0.05; *P* < 0.01; *P* < 0.01; *P* < 0.01, resp.). Nobiletin treatment gradually reversed the CUMS-induced deficit in sucrose intake, the onset of amelioration, that is a significant increase in sucrose preference as compared to CUMS-vehicle, was seen at week 2 and remained in following weeks treated with 40 mg/kg nobiletin (*P* < 0.05; *P* < 0.01; *P* < 0.01; *P* < 0.01, resp.). In addition, sucrose preference was reversed by nobiletin (20 mg/kg: *P* < 0.01, *P* < 0.01, resp.) and fluoxetine (10 mg/kg: *P* < 0.01, *P* < 0.01, resp.) at weeks 4 and 5.

As illustrated in Figure 1(b), the body weight of animals in each group does not have a significant difference at the beginning. A repeated ANOVA showed that during the 5-week experiment period, the body weight in the whole experiment showed a continual increase (*F*(5,140) = 56.82, *P* < 0.01). However, only fluoxetine increased the body weight compared with the CUMS-vehicle group at week 5 by post hoc test (*P* < 0.05). No significant differences were observed among the rest of the groups.

### 3.2. Effects of Nobiletin on the Serum Corticosterone Levels

**Figure 2:**
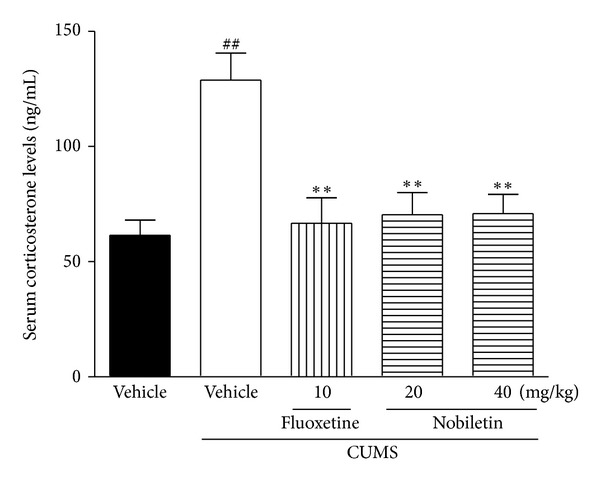
Effects of nobiletin on the serum corticosterone levels in CUMS-induced rats. The data represented the values of mean ± SEM (*n* = 8 in ELISA assay). ^##^ *P* < 0.01 versus control-vehicle group. **P < 0.01 versus CUMS-vehicle group.

The effects of nobiletin on the serum corticosterone levels in the CUMS rats were shown in Figure 2. The CUMS procedure caused a significant increase in serum levels of corticosterone in rats (*F*(1,14) = 24.98, *P* < 0.01). Compared with CUMS-vehicle animals, chronic treatment with 20 and 40 mg/kg nobiletin significantly reversed CUMS-induced elevation in corticosterone (*P* < 0.01, *P* < 0.01, resp.). In addition, the positive drug fluoxetine (10 mg/kg) also decreased the corticosterone levels in serum (*P* < 0.01).

### 3.3. Effects of Nobiletin on the BDNF Expression

**Figure 3:**
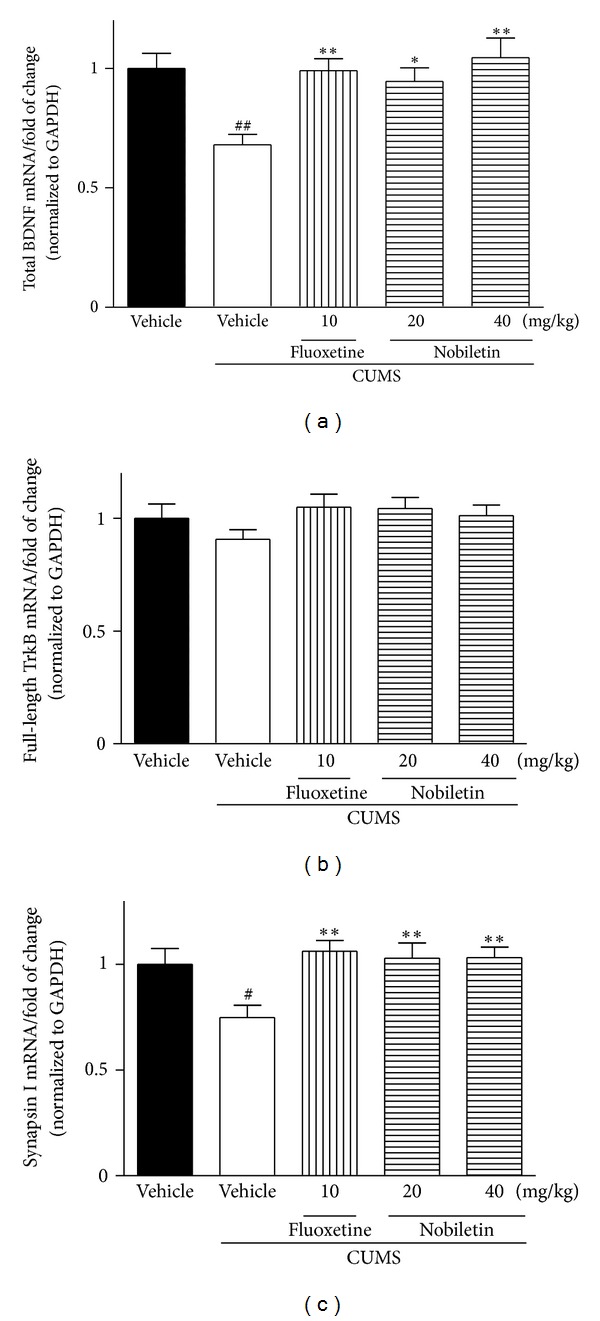
Effects of nobiletin on the hippocampal BDNF (a), TrkB (b), and synapsin I (c) mRNA expression in CUMS-induced rats. The data represented the values of mean ± SEM (*n* = 6 in PCR assay). ^#^ *P* < 0.05 and ^##^ *P* < 0.01 versus control-vehicle group. *P < 0.05 and **P < 0.01 versus CUMS-vehicle group.

**Figure 4:**
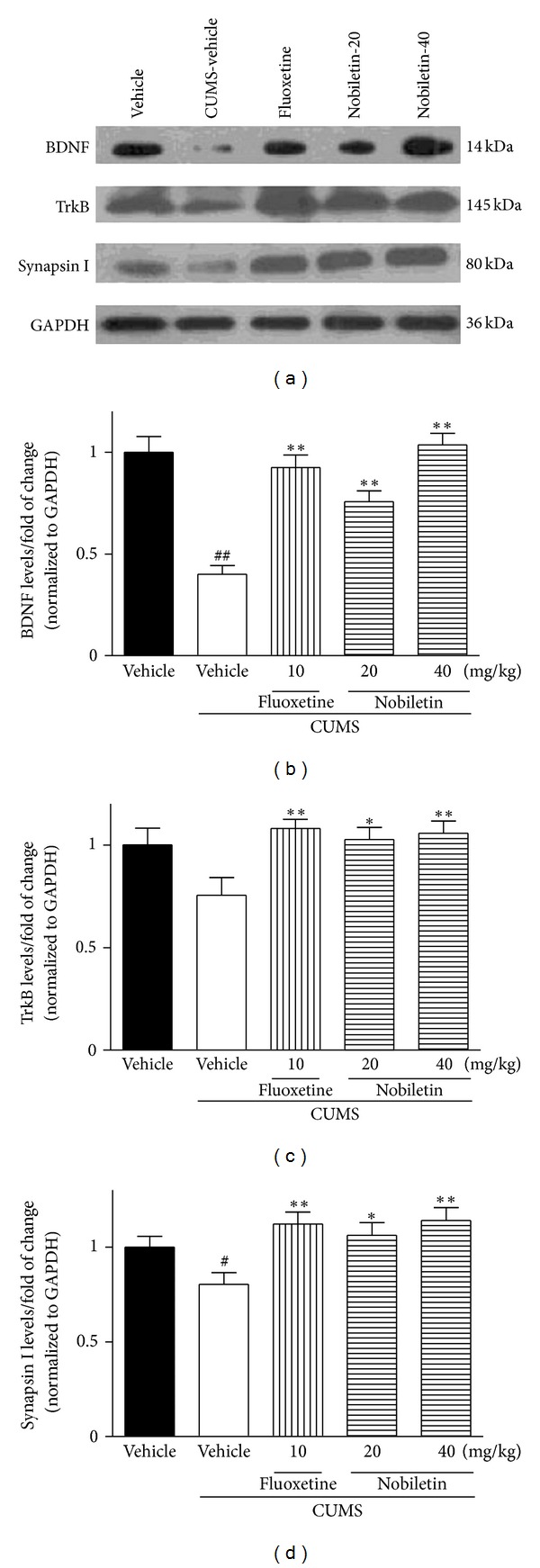
Effects of nobiletin on the hippocampal BDNF, TrkB, and synapsin I protein expression in CUMS-induced rats. (a) Representative western blot images of BDNF, TrkB, and synapsin I are shown, and BDNF (b), TrkB (c), and synapsin I (d) results were quantified and are the mean ± SEM (*n* = 6 in Western assay). ^#^ *P* < 0.05 and ^##^ *P* < 0.01 versus control-vehicle group. *P < 0.05 and **P < 0.01 versus CUMS-vehicle group.

The mRNA and protein expression of BDNF in the hippocampus were presented in Figures 3(a), 4(a), and 4(b), respectively. The levels of BDNF mRNA (*F*(1,10) = 17.51, *P* < 0.01) and protein (*F*(1,10) = 42.12, *P* < 0.01) in the hippocampus were significantly decreased by CUMS. Chronic nobiletin treatment at 20 (mRNA: *P* < 0.05; protein: *P* < 0.01, resp.) and 40 mg/kg (mRNA: *P* < 0.01; protein: *P* < 0.01, resp.) markedly elevated BDNF expression in the hippocampus. Fluoxetine also elevated BDNF mRNA and protein expression in this region (*P* < 0.01, *P* < 0.01, resp.).

### 3.4. Effects of Nobiletin on the TrkB Expression

In the hippocampus, TrkB mRNA and protein expression was slightly decreased (not statistically significant) by CUMS procedure compared with the control-vehicle group (Figures 3(b), 4(a), and 4(c)). Compared with CUMS-vehicle rats, chronic nobiletin treatment markedly elevated TrkB protein (*P* < 0.05, *P* < 0.01, resp.) but not mRNA expression. In addition, fluoxetine also increased hippocampal TrkB protein expression (*P* < 0.01) in CUMS animals.

### 3.5. Effects of Nobiletin on the Synapsin I Expression

The CUMS procedure significantly decreased the synapsin I mRNA (*F*(1,10) = 7.03, *P* < 0.05) and protein (*F*(1,10) = 5.50, *P* < 0.05) expression in the hippocampus (Figures 3(c), 4(a), and 4(d)). The synapsin I mRNA and protein levels were significantly increased in this region after treatment with nobiletin (mRNA: *P* < 0.01, *P* < 0.01; protein:*P* < 0.05, *P* < 0.01, resp.) or fluoxetine (*P* < 0.01, *P* < 0.01, resp.) in rats exposed to the CUMS procedure.

## 4. Discussion

As proposed by Willner, CUMS appears more suitable for studying the neurobiological basis of depression and the mechanisms of antidepressant agents, as compared to acute stress models such as FST or TST [17, 18]. Therefore, in the present study we investigated the effects of nobiletin on the hippocampal BDNF-TrkB system in the CUMS procedure. The CUMS animals exhibited a persistent reduction in responsiveness to pleasurable stimuli (a specific hedonic deficit), which is measured by a decrease in their sucrose preference, an indicator of anhedonia-like behavioral change in the CUMS [17]. CUMS-induced reduction in sucrose preference was also confirmed in our present study. As reported with conventional antidepressants [11, 25], chronic treatment with 20 and 40 mg/kg nobiletin caused a reversal of sucrose preference in rats exposed to CUMS. These results further confirmed the antidepressant-like actions of oral nobiletin administration in animal models of depression. Moreover, the results obtained from our study also indicated that nobiletin reversed the emerging anhedonia at week 2, suggesting that nobiletin produced rapidly acting antidepressant-like responses. However, there was a delay in the onset of the fluoxetine, since 4 weeks of fluoxetine treatment was needed for a complete recovery of anhedonia. Therefore, it suggests that nobiletin displayed a faster onset of action compared to the serotonin reuptake inhibitor fluoxetine. The rapid onset of nobiletin is supported by its rapid absorption in rodents [26, 27]. The concentration of nobiletin in rat brain increased to a maximum of 4.20 *μ*g/mL within 1.0 h after oral administration of 50 mg/kg nobiletin, and the mean area under curves (AUC_0–*t*_) in brain was 20.66 *μ*g·h/mL, which indicated that significant amount of nobiletin reached in brain rapidly. As a result, the pharmacokinetic profile of nobiletin supports that fast and significant brain permeability of nobiletin is enough to trigger rapid signal transduction cascades to produce therapeutic effects.

Since controversy exists regarding the influence of body weight change on sucrose consumption in the CUMS [28], it is important to measure this variable. Present study demonstrated that CUMS did not affect the body weight in the 5-week paradigm. Although some studies showed that CUMS caused a body weight reduction [11, 29, 30], several previous reports also demonstrated that CUMS exerted only slightly or no effect on body weight of rats [24, 31]. These results suggest some dissociation between body weight change and anhedonia in the CUMS [32], although body weight loss is also a diagnostic criterion for a clinical depression [33]. In addition, the effect of fluoxetine on body weight is still controversial. Our data displayed that fluoxetine but not nobiletin increased the body weight compared with CUMS-vehicle group. However, no significant effect of fluoxetine on body weight was shown in another preclinical study [11]. These discrepancies may be produced by many parameters in the experimental design, mainly including strains of animals, experimental conditions, and stressed time [34]. On the other hand, according to some preclinical reports, nobiletin can induce fatty acid oxidation, suppress adipocyte differentiation, and enhance adiponectin secretion and lipolysis, which resulted in reduction of body weight gain [35–37]. Therefore, the inability of nobiletin to produce body weight gain could be explained by that the restoration of eating behavior (anhedonia improvement) is counteracted by increased lipolysis.

Multiple mechanisms are responsible for the development of depression. The hypothalamic-pituitary-adrenal (HPA) axis is involved in the pathogenesis of depression and plays a key role in mediating the responses to various stressful stimuli and the effects of the antidepressant [38]. The exposure to an acute stressor activates the HPA axis, resulting in a cascade of endocrine events. This cascade finally stimulates the production and release of glucocorticoids [39]. The hippocampus has very high levels of glucocorticoid receptor (GR) and provides negative feedback to the hypothalamus to prevent further release of glucocorticoids [40]. However, once the stress sustains, glucocorticoid hypersecretion will reduce GR expression and impair negative feedback inhibition. Subsequently, hippocampus will be damaged, reduced in size or functionally suppressed [41]. As a result, further harmful processes will be done, resulting in cognitive deficits, decreased appetite, and behavioral anhedonia [42]. Corroborated by observations from our previous and other studies [2, 25, 43], the present study exhibited that the HPA hyperactivity induced by CUMS, as indicated by an increased serum corticosterone level, was ameliorated by nobiletin and fluoxetine administration for 5 weeks. The finding suggested that the antidepressant-like actions of oral nobiletin treatment might be partly related to the modulation of the HPA axis activity. Studies of both serotonergic and noradrenergic antidepressants suggested that successful treatment normalized the measures of the HPA axis function in depressed patients and stressed animals [44, 45]. These antidepressants improved hippocampal function, stimulated GR expression, and then restored the negative corticosteroid feedback [46]. As a result, the normalized negative feedback reduced serum corticosterone levels mediated through corticotropin-releasing hormone/adrenocorticotropic hormone inhibition [47]. Based on our previous report that nobiletin produced an antidepressant-like effect through serotonergic, noradrenergic and dopaminergic, systems [21], we speculate that the action of nobiletin on the HPA axis is, thus, likely to be exerted via the monoaminergic systems. However, it should be noted that the mechanism of nobiletin to inhibit serum corticosterone secretion is complicated, since glucocorticoid is affected by many factors, and there is no consistent evidence of a simple relationship between the monoaminergic system and HPA axis [48]. Therefore, there would be a dual system for the HPA restoration: directly, via unknown mechanisms (such as cytokines), and via monoaminergic systems [49, 50].

In recent years, the biological research of depression has moved beyond the monoamine hypothesis [23]. Neurotrophins and their receptors are also involved in the function of the hippocampus, where some of them, in particular BDNF and TrkB receptor, are widely expressed [51]. Increasing clinical and experimental evidence indicated that BDNF played a role in the pathophysiology of depression and that antidepressants may in part exert their effects through regulation of BDNF [7]. For example, downregulation of BDNF expression was found in hippocampal region from depressed suicide victims relative to matched controls in a clinical study [52]. Many of the chronic stressors, such as CUMS or social defeat stress, have also been found to robustly downregulate hippocampal BDNF mRNA and protein expression in rodents [11, 53]. Consistently, data displayed in our study also indicated that 5-week CUMS significantly decreased the hippocampal BDNF mRNA and protein expression. In contrast to the effects of CUMS, nobiletin and fluoxetine treatments restored the reduction of BDNF expression in the model, which was partly consistent with a previous study that nobiletin possessed neurotrophic action in PC12 cells [54]. 

In parallel with BDNF, increased mRNA expression of hippocampal TrkB receptor is involved in the therapeutic action of antidepressant treatment in rats [55]. TrkB receptor, as well as BDNF in hippocampus, is decreased in depressed patients [56]. Chronic antidepressant treatment has been reported to enhance hippocampal neurogenesis as well as to increase the expression of BDNF and TrkB receptor in animal models [7, 57]. BDNF initiates TrkB receptor-dependent different intracellular signaling pathway and exhibits beneficial effect for the treatment of depression in experimental studies [58]. Although BDNF-TrkB signaling is downregulated in hippocampus according to animal models of stress and/or depressive-like behavior [59, 60], some studies failed to detect evidence for depression with reduced BDNF levels in meta-analyses [61] or heterozygous mutant *bdnf* ^+/−^ mice [62]. However, in contrast to the unclear association with the pathophysiology of depression, the neurotrophin signaling appears to be required for antidepressant activity. In a previous study, drugs that block TrkB signaling pathway can break the antidepressant-like effect of BDNF in rats [59]. In contrast to our data on BDNF mRNA expression, hippocampal TrkB mRNA expression did not modify after the 5-week CUMS procedure or drug treatments. However, TrkB protein expression increased following nobiletin or fluoxetine treatment, although the inhibitory effect of CUMS did not reach a significant level. This finding suggests that nobiletin may affect the rate of TrkB protein synthesis or degradation but not TrkB mRNA. Although the mechanism of nobiletin-induced TrkB protein upregulation is unknown, the role of ubiquitin-proteasome pathway has been suggested for ligand-induced TrkB degradation [51, 60]. Further studies are necessary to determine whether ubiquitin system is involved in nobiletin-induced TrkB regulation. In addition, it should be noted that a recent study found that 5 mg/kg fluoxetine treatment administration for 5 weeks did not alter expression of hippocampal TrkB protein in rats [24]. The reason for this discrepancy may be due to different time points at which TrkB expression was assessed after the last exposure to the stressor [63]. In their study, after 5-week procedure of CUMS, the animals were submitted to behavioral tests for two weeks and sacrificed at week 7 [24]. Another reason could possibly be attributed to the different doses and administration routes of fluoxetine used.

In addition to increasing BDNF levels and TrkB signaling, antidepressants also increase its downstream target of synaptic protein in rodents [64, 65]. For example, fluoxetine-induced changes in synaptic protein expression were mediated by TrkB signaling in rats [12]. Synapsins are the most abundant synaptic proteins and play multiple roles in synaptic transmission and plasticity [66]. Reduction of synapsin in the hippocampus was found in patients with mental disorder [67]. CUMS procedure has been demonstrated to alter brain structure and function in rat, causing suppression of synaptic plasticity in the hippocampus [68]. Collectively, since BDNF-TrkB signaling pathway can mediate behavioural responses to antidepressants and induce neuronal plasticity, we investigated the contribution of the signaling to nobiletin-induced changes in synapsin I expression, one of the major synaptic vesicle-associated proteins. Consistent with previous studies [69, 70], CUMS suppressed the expression of synapsin I at both the mRNA and protein levels. Chronic nobiletin and fluoxetine completely abolished this CUMS-induced deficit. This finding suggests that nobiletin exerts downstream effects on synapsin I similar to those of classical antidepressant fluoxetine.

In addition, glucocorticoids play a critical role in mediating stress-induced downregulation of BDNF in the hippocampus in animals [63]. Repeated corticosterone administration, like stress, decreased the BDNF levels in the hippocampus of normal or adrenalectomized rats [71, 72]. A significant hypersecretion of corticosterone with downregulation of hippocampal BDNF was also found in the GR-deficient mice [73]. In contrast, there was a significant induction in BDNF expression in rats after removal of the adrenal glands [74]. Moreover, BDNF-enriched synaptic protein of synapsin I was suppressed by glucocorticoid *in vitro* [54]. Together with the literatures, we speculated that BDNF-TrkB pathway restoration by nobiletin may be mediated by corticosterone.

Taken together, the results of the present work showed that anhedonic behavior, as well as downregulation of hippocampal BDNF, TrkB, and synapsin I expression was observed in rats subjected to CUMS. The results also suggested that nobiletin counteracted the ability of CUMS to induce depression-like behavior in rats and might involve maintenance of hippocampal BDNF-TrkB pathway. However, nonstressed rats treated with nobiletin did not adopt in our present study, data concerning behavioral and biochemical changes in nonstressed rats would certainly provide some additional explanations on the antidepressant-like mechanism of nobiletin; so this is one of the limitations of the present study. Finally, since enhanced induction expression of BDNF in response to chronic antidepressant agent treatments could promote neuronal survival and protect neurons from the damaging effects of stress in rat brain [36], BDNF/TrkB-dependent neurogenesis involved in the antidepressant-like mechanism of nobiletin will be further investigated by using inhibitors/activators or gene block-out/silence technique.

## Figures and Tables

**Table 1:** Time and length of stressors used in the CMS procedure. These stressors which were applied continuously, were randomly scheduled over a 1-week period and repeated throughout the 5-week period. At the same time, animals were treated with nobiletin (20 and 40 mg/kg, P.O.), fluoxetine (10 mg/kg, P.O.), or saline.

	Monday	Tuesday	Wednesday	Thursday	Friday	Saturday	Sunday
Food and water deprivation	16:00→10:00			08:00–20:00			
Stroboscopic illumination		12:00–20:00				20:00→08:00	
White noise		20:00→08:00					08:00–20:00
Light/dark succession every 2 h			08:00–20:00			08:00–20:00	
Overnight illumination			20:00→08:00				
45° cage tilt	→16:00			20:00→08:00			20:00→
Soiled cage					08:00–20:00		
Pair-housing					20:00→08:00		
Sucrose preference test		10:00–12:00					
